# The pattern of non-contact injuries in a South African professional football team

**DOI:** 10.17159/2078-516X/2022/v34i1a13723

**Published:** 2022-01-01

**Authors:** J Swart, C Varekamp, J Greyling

**Affiliations:** 1 HPALS Research Centre, Faculty of Health Sciences, University of Cape Town, South Africa; 2 Point Physiotherapy, Cape Town, South Africa

**Keywords:** football injuries, training load, epidemiology

## Abstract

**Background:**

The incidence, pattern and severity of non-contact injuries in European football has been researched extensively. In South African football only two studies have been conducted to date and with disparate outcomes. Further research into injury rates in South African football is therefore warranted.

**Objectives:**

To determine the incidence and pattern of non-contact injuries in a South African professional football team during the course of a single season (2016–2017) in relation to competition exposure, training load and playing position.

**Methods:**

Thirty-four male professional football players belonging to a single team competing in the Premier Soccer League (PSL) in South Africa were studied. Non-contact time-loss injuries (total training and match injuries) were recorded. Injury incidence, location, severity, type, and playing position (defender, midfielder, attackers, goalkeepers) during either match play or training were recorded.

**Results:**

The non-contact incidence was 52 injuries with an injury rate of 3.74 per 1 000 exposures (training and competition). Competitions resulted in an incidence of 26.4 injuries per 1 000 exposure and training incidence 2.08 injuries per 1 000 exposures. Hamstring, groin and quadriceps injuries were the most frequently injured locations and muscle-tendon injuries accounted for the majority of injuries. The majority of injuries (52%) occurred during match play while 48% occurred during training. The greatest absolute number of injuries were sustained by midfielders (50%), followed by defenders (33%) and attackers (17%). However, relative to player numbers, the greatest number of injuries during match play were for defenders (44%), attackers (32%) and midfielders (24%). During training attackers sustained the most injuries (39%), followed by defenders (31%) and midfielders (30%). Goalkeepers did not sustain any non-contact injuries during the duration of the study.

**Conclusion:**

The non-contact injury incidence in South African professional football players is similar to European football players. Hamstrings and groin injuries are predominant and were sustained throughout the competitive season. Defenders sustained the most non-contact injuries within the team relative to exposure time compared to attackers and midfielders. To our knowledge, injuries relative to player position have not been reported previously.

Football has grown into a faster, more intense and more competitive game, with a substantial increase in technical and physical demands resulting in a higher risk of injuries. ^[1]^ This risk is significantly higher during match play compared to training. ^[2]^ Norwegian football players sustained 1.4 non-contact injuries per 1 000 exposures and this risk was not significantly higher during pre-season compared to the rest of the competitive season. ^[3]^ European football players sustain on average 0.6 muscle-related injuries per season, which results in 15 muscle-related injuries per team per season. ^[4]^ A recent meta-analysis of football-related injuries in professional players showed an overall incidence of 8.1 injuries per 1 000 exposure hours, a training incidence of 3.7 injuries per 1 000 exposures and a match incidence of 36 injuries per 1 000 hours of match exposure. Non-contact injury rates were 2.1 per 1 000 exposures. ^[5]^ Current data recorded for South African football are scarce, with one study recording an overall injury rate of 2.2 per 1 000 exposures ^[6])^ while another recorded 13.4 injuries per 1 000 exposures. ^[7]^ Further research into injury rates in South African football is therefore warranted.

Of all injuries sustained, non-contact injuries account for 9–35%. ^[3]^ The lower extremity is the most commonly affected site in professional football players. ^[6]^ This is due to the continual load placed on the legs during training and competition. The hamstrings are the most commonly affected muscles, accounting for 37% of injuries, followed by adductors (23%), quadriceps (19%) and calf muscles (13%).^[4]^

To reach the peak demands of match play, extensive training is necessary to improve performance and to reach the top level in professional football. Inadequate training loads prevent optimal performance adaptions, placing the player at higher risk of being underprepared and may increase the risk of non-contact injuries.^[8]^ Inadequate training load (TL), repetitive movements, deficient technical execution performed for long periods in combination with inadequate and insufficient recovery or rehabilitation are possible causes of non-contact injuries. ^[9,10]^

In addition, playing position has a large influence on physiological demands and this may influence the risk of sustaining an injury. ^[1,11]^ Different positional roles require unique physiological, technical and tactical performances from the individual player.^[1]^ Leventer^[11]^ determined that midfielders sustain the most injuries (38%) and have the highest incidence of match injuries, followed by defenders (30%) and attackers (21%). However this finding has not been confirmed by other researchers ^[12]^, while the injury risk relative to the number of players in each playing position or the injury risk relative to the number of exposures in each position of play has not been examined directly.

We documented the incidence of non-contact injuries in a professional football team during a competitive football season. In addition, injury rate per player position and position specific exposure were documented.

## Methods

### Participants

Thirty-four male professional football players belonging to the first team of a professional football team ((mean ± SD): age 24.3 ± 4.1 years; height 173.4 ± 6.0 cm; body mass 74.3 ± 6.7 kg) were recruited during the 2016/2017 competitive South African Premier Soccer League (PSL) season. The players were recruited through direct communications with the club. All participants provided written informed consent and the research study was approved by the University of Cape Town Human Research Ethics Committee.

### Procedures

Participants were enrolled in the study for 11 months. Exposure data were recorded for each player for all training sessions and matches by the conditioning staff. All injuries were assessed and recorded by the medical staff of the team. These data included the date and time of the injury, mechanism of injury, location, type of injury, severity, recurrence of injury and duration before return to play (RTP).

### Data analysis

The analysis included all non-contact injuries sustained during all on-field training sessions and matches throughout the 2016/2017 competitive season. A non-contact injury was defined as an injury occurring suddenly or insidiously and progressively over time and presumably caused by repetitive motion or micro trauma and/or accumulative movements without an identifiable responsible event such as a collision. ^[9,13]^ Non-contact injuries differ from acute contact injuries caused by an acute moment or identifiable trauma.

Injuries were defined as either training or match related and were recorded as time-loss injuries if the player was not able to return to play within 24 hours.^[14]^ Medical attention injuries whereby the player was able to participate in full training the next day (within 24 hours) were not included in this research. A recurrent injury was defined as an injury affecting the same structure, at the same site and of the same type as the previous injury, which occurred after return to play ^[14]^, during the course of the 2016/2017 competitive season.

Injuries unrelated to football, contact injuries and illness were not included in this research. The study therefore only included time-loss non-contact injuries.

Injuries were categorised according to the site of injury and included; hip, groin, adductors, quadriceps, hamstrings, knee, calf, and ankle, and foot.

Injury rates were assessed as the number of non-contact injuries occurring relative to each 1 000 training and match play hours (exposures). Groin injuries were classified according to the Doha consensus agreement ^[15]^, and included adductor, iliopsoas, inguinal, hip, and pubic-related groin pain.

Injuries were ranked as minimal, mild, moderate, and severe based on days out of training and competition. A minimal injury was defined as >24 hours – 3 days of football activity missed, mild (4–7 days), moderate (1–4 weeks) or severe (4+ weeks). ^[14]^ The risks of these injuries are calculated as the number of injuries sustained relative to the exposure of each workload classification.

### Injury exposure and incidence

Injury incidence was calculated by dividing the total number of non-contact injuries by the overall injury exposure, and expressed as rates per 1 000 hours. ^[13]^

Match injury exposure was calculated by multiplying the number of players by the session duration of match play and the number of matches played. ^[13]^ Total number of non-contact injuries sustained during match play were divided by the exposure hours and described as injuries per 1 000 match playing hours ^(13)^.

On-field training non-contact injury exposure was calculated by multiplying the number of players by the average duration of the on-field training sessions and the number of on-field training sessions during the full competitive season. ^[13]^ The team also performed, on average, two strength training sessions per week of 60 minutes duration each and these were included in the training exposure rates. The total number of injuries sustained during training sessions was therefore divided by the total exposure hours of on-field training and strength training and described as injuries per 1 000 training hours. ^[13]^

Injury statistics were grouped by player's predominant position in the team. Relative injury exposure was also calculated per number of players per playing position and per hours of training and match play.

## Results

### Injury incidence

**Fig. 1:**
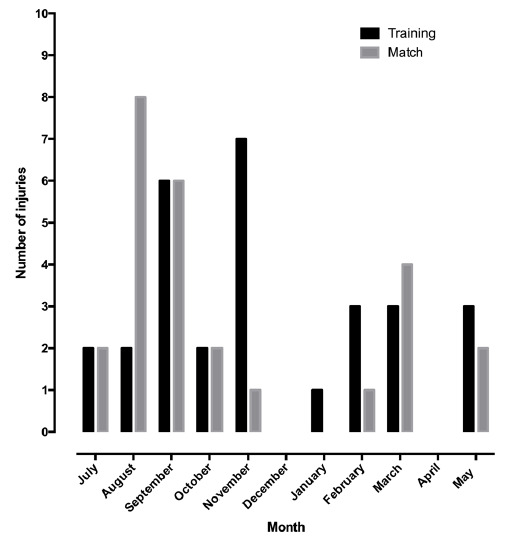
Injuries sustained during training or match per month

Fifty-two non-contact injuries were sustained throughout the season, resulting in 3.74 non-contact injuries per 1 000 hours of exposure. Twenty-seven non-contact injuries (52%) were sustained during match play, resulting in 24.8 injuries per 1 000 hours of match play. Twenty-five non-contact injuries (48%) were sustained during training, resulting in 1.96 injuries per 1 000 hours of training (Figure 1).

Descriptive statistics for all overuse injuries are summarized in Table 1.

### Injury site

Muscles were the most frequently injured structures (85%). Hamstring strains accounted for 48% of the total non-contact injuries sustained. Groin injuries including adductor, inguinal and pubic-related groin pain accounted for 23% of all injuries sustained. Quadriceps injuries were the third most common site of injury (12%). Others, defined as injuries affecting hip flexors and gluteal muscles accounted for 6% of all injuries. Time-loss injury affecting the hip joint, specifically a hip labral injury only occurred once (2%). Lower leg injuries, including gastrocnemius and peroneus longus injuries, occurred three times (6%). Non-contact overuse injuries affecting both the ankle and the knee (4%) only occurred once.

The majority of hamstring (56%), quadriceps (66%) injuries occurred in the first 3 months of the season and overall 46% of the injuries occurred during this period. In contrast, groin injuries were mostly sustained in the mid-season and late season. Lower leg injuries occurred in the pre-season and the end of the season.

### Position injury risk

Midfielders were most likely to sustain an injury (50%), followed by defenders (32.7%) and attackers (17%). However, when expressed as injuries per 1 000 exposure hours, defenders sustained 23.7 injuries per 1 000 hours of match play (44% of all injuries). Attackers sustained 17.2 injuries per 1 000 hours of match play (32%). Midfielders sustained 10.7 injuries per 1 000 hours of match play (24%). During training, attackers were most likely to be injured and sustained 2.12 injuries per 1 000 hours (39% of all injuries). Defenders sustained 1.7 injuries per 1 000 hours (31%) and midfielders only sustained 1.6 injuries per 1 000 hours (30%). Goalkeepers did not sustain any non-contact injuries.

**Fig. 2:**
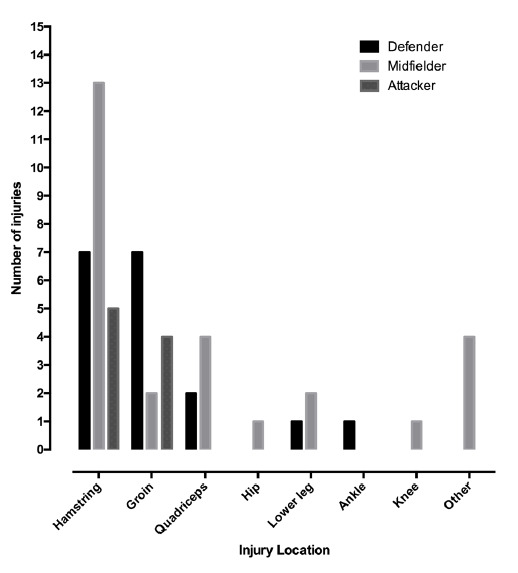
Overuse injuries relative to playing position

Midfielders experienced the highest risk of absolute injuries affecting the hamstring, quadriceps, hip flexors and knee. Defenders sustained mostly hamstring and groin injuries. Attackers were least often injured, but if injured, usually suffered hamstring, groin and lower leg injuries (Figure 2).

## Discussion

The objective of this study was to describe the pattern of non-contact injuries in South African professional football players. A total of 52 overuse injuries were analysed in this study.

A previous study of professional South African football players by Calligeris, Burgess, and Lambert^[7]^ reported 130 injuries from all causes over a full competitive season. In contrast to our study, Calligeris et al ^[7]^ reported all injuries sustained, including contact injuries and injuries affecting the upper extremity. The study reported an overall injury rate of 13.4 injuries per 1 000 playing hours. In contrast, when only assessing non-contact injuries, we found an injury rate of 3.74 per 1 000 hours, which is similar to the reported incidence of 2.70–8.70 injuries per 1 000 playing hours on average in studies of European football players. ^[4]^ In addition, we found a significant difference between non-contact injuries between matches and training with 24.8 non-contact injuries per 1 000 match hours and 1.96 overuse injuries per 1 000 hours of training. Fuller ^[16]^ investigated all cause injury incidence of English Premier League football clubs and reported a match injury incidence of 26.9 per 1 000 hours and a training injury incidence of 4.3 per 1 000 hours. This incidence is only marginally greater than our data, partly due to the fact that Fuller included all injuries.

Twenty-five of our reported injuries were sustained during training and 27 overuse injuries were sustained during match play. This similar to the findings in the study by Lu et al. ^[17]^, who reported that 60%^[4]^ of all non-contact injuries were sustained during match play. Out of the 27 non-contact injuries sustained during match play in our study, 17 of these injuries affected the hamstring and six affected the groin. Several other studies ^[4,8,11,14]^ have also concluded that hamstrings and groins are most often injured during match play.

We reported that hamstring and quadriceps injuries were mostly sustained in the first three months of the competitive season. Although only one official league match was played, five friendly matches were played during this time. This supports the hypothesis of Gabbett & Domrow^[18]^, that the training load in the pre-season period is the greatest and training injuries in pre-season are therefore unavoidable. The study by Impellizzeri^[19])^ also concluded that friendly matches were more regularly played in pre-season compared to in-season, which resulted in different periodisation and overall training load during the full season. This is also in line with the study by Murray^[20]^ that reported relatively more injuries in the pre-season compared to in-season. It may be assumed that an appropriate fitness level is reached in the second half of the season, therefore resulting in a lower number of overuse injuries.

Perhaps for the first time, we have described injury risk for player position according to the number of exposures for that position. This demonstrates that although the injury rate for midfielders is generally reported as being greatest, this may be due to the higher number of players in a squad who play in the midfield. When expressed relative to exposures, defenders are most likely to be injured during matches and attacking players are most likely to be injured during training. More research is required to confirm these findings and to elaborate on other factors which may contribute to this pattern.

## Conclusion

Non-contact injuries in South African football occur at a similar incidence to European counterparts. Most overuse injuries occur during matches and within the first three months of the season. Hamstring and groin injuries are the most common injuries sustained. Although midfielders sustain the most injuries, when expressed relative to exposure hours, defenders sustain the greatest number of injuries.

## Figures and Tables

**Table 1:** Total overuse injuries 2016/17 full competitive season (n=52)

Injury classification	Number	%
**Activity performed**		
Training	25	48
Match	27	52

**Affected body part**		
Hamstring	25	48
Groin	12	23
Adductor related groin pain	6	12
Pubic related groin pain	4	8
Inguinal related groin pain	2	4
Quadriceps	6	12
Others (hip flexor and glut.)	3	6
Hip	1	2
Lower leg	3	6
Ankle	1	2
Knee	1	2

**Severity**		
Minimal	5	10
Mild	13	25
Moderate	30	58
Severe	4	8

**Position played**		
Defenders	17	33
Midfielders	26	50
Attackers	9	17
Goalkeepers	0	0

Data rounded off to the nearest whole number.
